# Effects of candesartan in hypertensive patients with type 2 diabetes mellitus on inflammatory parameters and their relationship to pulse pressure

**DOI:** 10.1186/1475-2840-11-118

**Published:** 2012-10-03

**Authors:** Masaya Sakamoto, Hirofumi Suzuki, Takeshi Hayashi, Hiroyuki Iuchi, Tsuyoshi Isaka, Noriko Sakamoto, Yosuke Kayama, Katsuyoshi Tojo, Michihiro Yoshimura, Kazunori Utsunomiya

**Affiliations:** 1 Division of Diabetes, Metabolism and Endocrinology, Department of Internal Medicine, Jikei University School of Medicine, 3-25-8 Nishi-Shinbashi, Minato-ku, Tokyo, 105-8461, Japan; 2 Division of Cardiology, Department of Internal Medicine, Jikei University School of Medicine, Jikei University School of Medicine, 3-25-8 Nishi-Shinbashi, Minato-ku, Tokyo, 105-8461, Japan

**Keywords:** Candesartan, Angiotensin receptor blockers, Type 2 diabetes mellitus, Inflammatory parameters, Pulse pressure

## Abstract

**Background:**

Angiotensin receptor blockers (ARBs) are reported to provide direct protection to many organs by controlling inflammation and decreasing oxidant stress in patients without arteriosclerosis. This study aimed to evaluate (1) whether an ARB (candesartan) decreases values for inflammatory parameters in hypertensive patients with type 2 diabetes mellitus of long duration accompanied by arteriosclerosis and (2) whether there any predictors of which patients would receive the benefits of organ protection by candesartan.

**Methods:**

We administered candesartan therapy (12 mg daily) for 6 months and evaluated whether there was improvement in serum inflammatory parameters high molecular weight adiponectin (HMW-ADN), plasminogen activator inhibitor-1 (PAI-1), highly sensitive C-reactive protein (Hs-CRP), vascular cell adhesion molecule-1 (VCAM-1) in serum and urinary-8-hydroxydeoxyguanosine (U-8-OHdG). We then analyzed the relationship between the degree of lowering of blood pressure and inflammatory factors and the relationship between pulse pressure and inflammatory factors. Finally, we analyzed predictive factors in patients who received the protective benefit of candesartan.

**Results:**

After 6 months of treatment, significant improvements from baseline values were observed in all patients in HMW-ADN and PAI-1 but not in Hs-CRP, VCAM-1 and U-8-OHdG. Multilinear regression analysis was performed to determine which factors could best predict changes in HMW-ADN and PAI-1. Changes in blood pressure were not significant predictors of changes in metabolic factors in all patients. We found that the group with baseline pulse pressure <60 mmHg had improved HMW-ADN and PAI-1 values compared with the group with baseline pulse pressure ≥ 60 mmHg. These results suggest that pulse pressure at baseline could be predictive of changes in HMW-ADN and PAI-1.

**Conclusions:**

Candesartan improved inflammatory parameters (HMW-ADN and PAI-1) in hypertensive patients with type 2 diabetes mellitus of long duration independent of blood pressure changes. Patients with pulse pressure <60 mmHg might receive protective benefits by candesartan.

**Trial registration:**

UMIN000007921

## Background

Almost half of type 2 diabetic patients are reported to have hypertension during their lifetime [1], and the coexistence of hypertension and diabetes mellitus especially increases the risk of cardiovascular events. Moreover, much of this excess risk is attributable to coexistent hypertension [2]. Treatment of hypertension in patients with type 2 diabetes mellitus of short duration has been shown to reduce cardiovascular events [3,4]. Therefore, in addition to glycemic control, the treatment of hypertension is important in preventing cardiovascular events. On the other hand, unexpectedly, the ACCORD BP study recently performed in patients with type 2 diabetes of long duration (average 10 years) showed that strict blood pressure control failed to reduce cardiovascular events [5]. But a detailed examination of that result, which included investigation of inflammatory parameters, has not been performed.

Angiotensin receptor blockers (ARBs) are regarded as first line therapy for hypertensive patients with type 2 diabetes mellitus [6,7]. In addition to their antihypertensive effects, results of a multi-center trial showed that ARBs had a role in preventing the development of type 2 diabetes [8]. Also, ARBs have been shown to protect many organs by controlling inflammation [9-12] and decreasing oxidant stress [13] in hypertensive patients without arteriosclerosis. But there is little evidence that ARBs have a positive effect in patients with advanced arteriosclerosis, such as those with type 2 diabetes mellitus of long duration accompanied by hypertension as were the patients in the ACCORD BP study. Furthermore, pulse pressure increases during the process of arteriosclerosis. It has been reported that pulse pressure is not only a predictor of cardiovascular events [14,15], but also is an independent predictor of new-onset diabetes in high-risk hypertensive patients [16].

In this study, we administered an ARB (candesartan: only sartan which can use for chronic heart failure patients in Japan) to hypertensive patients with type 2 diabetes mellitus and evaluated whether there was improvement in the following inflammatory parameters: high molecular weight adiponectin (HMW-ADN), plasminogen activator inhibitor-1 (PAI-1), highly sensitive C-reactive protein (Hs-CRP), vascular cell adhesion molecule-1 (VCAM-1) and urinary 8-hydroxydeoxyguanosine (U-8-OHdG). We then analyzed the relationship between the degree of lowered blood pressure and values for inflammatory factors and the relationship between pulse pressure and pulse wave velocity (PWV), which are index parameters of arteriosclerosis. Finally, we analyzed predictors of patients who would receive benefit from candesartan by protecting their organs.

## Methods

### Participants

This was a prospective study. Patients were targeted for enrollment among hypertensive patients with type 2 diabetes mellitus who regularly attended the Jikei University School of Medicine affiliated hospital for treatment. We enrolled 56 patients (46 males and 10 females, 25–75 years old, average 60) who had hypertension (defined as diastolic blood pressure [DBP] ≧80 mmHg or systolic blood pressure [SBP] ≧130 mmHg, average 138/79) or were taking antihypertensive medicine (Table 1). Patients with secondary hypertension were excluded, as were patients with impaired liver function defined as plasma aminotransferase (or aspartate aminotransferase) over 39 mUml (normal values: 11–39 mUml) and alanine aminotransferase over 34 mUml (normal values: 11–34 mUml) or impaired kidney function (defined as serum creatinine level over 1.3 mg per 100 ml (normal values: 0.6–1.3 mg per 100 ml). Patients with unstable cardiovascular conditions (e.g., New York Heart Association class I–IV congestive heart failure or a history of myocardial infarction or stroke) or cerebrovascular incidents within 6 months of study enrollment were also excluded. Women who were pregnant, lactating, or who might become pregnant due to inadequate contraceptive precautions were also excluded. Patients with known contraindications or intolerance to candesartan were also not included in the study.

**Table 1:** Baseline data on study subjects

N	56
Sex (M/F)	46/10 (82.1%)
Age (y)	60.84
BMI (kg/m^2^)	25.07
Blood pressure(SBP/DBP) (mmHg)	138 (9.67)/78.6 (11.5)
Dulation (y)	15.47 (10.67)
Laboratory data	
HbA1c (%)	6.87 (0.68)
FPG (mg/dl)	151.7 (47.5)
LDL-C (mg/dl)	109.7 (25.3)
HDL-C (mg/dl)	55.7(12.8)
Triglycerides (mg/dl)	150(83.0)
Creatinine (mg/dl)	0.80 (0.18)
Blood glucose lowering treatment	
Insulin only	9
Insulin+OHA	6
OHA	31
Diet only	6
Dropped out	10

F, female; M, male, BMI, Body-mass index; HbA1c, glycated hemoglobin; FPG, fasting plasma glucose; LDL-C, Low density lipoprotein-cholesterol; HDL-C, High density lipoprotein-cholesterol; OHA, oral hypoglycemic agent, *P<0.05. Data are expressed as means±s.d. or n and%.

Patients were administered 12 mg candesartan daily at the same time for a duration of 6 months. At the beginning of the study, if patients were taking an ARB or ACE-I, that drug was replaced with 12 mg candesartan. Antihyperglycemic drugs were not changed during this study.

The study protocol was approved by the institutional review board at Jikei University School of Medicine and conducted in accordance with the Declaration of Helsinki and its amendments. After a full explanation of the study, all patients gave written informed consent.

### Assessment of participants

Before starting the study, all patients underwent an initial screening assessment that included a medical history and physical examination. We evaluated patients at the start of the study to establish baseline values, then again after the 6^th^ month of treatment. Parameters were as follows: body weight, body mass index (BMI), SBP, DBP, PWV, HbA1c, fasting plasma glucose (FPG), HMW-ADN, PAI-1, Hs-CRP, VCAM-1 and U-8-OHdG.

To evaluate tolerability to candesartan, all adverse events were recorded. All plasmatic parameters were measured after a 12-h overnight fast. In all cases, venous blood samples were taken between 800 and 900 h. We used plasma obtained by the addition of Na2-EDTA (1mgml_1) and centrifuged at 3000 g for 15 min at 4°C. All measurements were performed in a central laboratory.

BMI was calculated as weight (kg) divided by the square of height (m). Height and weight were determined using a standard scale (SYSTEM 502, TANITA, Tokyo, Japan). Aortic PWV measurements using the right carotid and right femoral arteries were performed with the patient in a supine position after resting at least 5 min. A Vasera VS-1500A System (Fukuda Denshi, Tokyo, Japan) device was used at each site. All personnel were trained and certified to take blood pressure measurements from the right arm with a Tyco aneroid sphygmomanometer using American Heart Association standards and to perform the aortic PWV measurements. Blood pressure measurements were obtained from the patients’ right arm while they were in a seated position, using a standard sphygmomanometer (ADVANCE BP-203RVIIIC/D, OMRON colin, Tokyo, Japan) (Korotkoff I and V) with an appropriately sized cuff. Furthermore, the same investigator measured patients’ blood pressure at each visit, always in the morning and after the patient had rested for at least 10 min in a quiet room. Three successive blood pressure readings were obtained at 1-min intervals, and the mean of the three readings was calculated. HbA1c level was measured by a high-performance liquid chromatography method (HLC723-G9, TOSOH, Tokyo, Japan; normal values Japan Diabetes Society: 4.4–5.8%), with intra- and inter-assay coefficients of variation (CsV) of 1%. Plasma glucose was assayed by the glucose-oxidase method (GA08II, A&T, Yokohama, Japan) with intra- and inter-assay CsV of 0.8%.

Plasma HMW-ADN level was determined using an CLEIA (chemiluminescent enzyme immunoassay; Fuji Rebio,Tokyo, Japan). Plasma VCAM-1 level (normal values 277-836 ng/ml) was determined using an ELISA (enzyme-linked immunosorbent assay; R&D Systems. Inc. Minneapolis, U.S.A). Plasma PAI-1level (normal values ≤50 ng/ml) were determined using a LPIA (latex photometric immunoassay; Mitsubishi Chemical Medience. Tokyo, Japan). The U-8-OHdG level (normal values: 6.1-16.3 ng/mg · cr) was measured using HPLC (high performance liquid chromarography; Mitsubishi Chemical Medience. Tokyo, Japan). Plasma hs-CRP level (normal values ≤0.3 mg/dl) was measured using the latex agglutination nephelometry method (Siemens Healthcare Diagnostics Inc. Marburg, Germany).

### Statistical analysis

Statistical analysis of data was performed using the Statistical Package for Social Sciences software, version 19.0 (SPSS, Chicago, IL, USA). The data are presented as the mean±s.e. For all statistical analyses, P<0.05 was considered statistically significant.

## Results

### Characteristics of study sample

Fifty-six patients were enrolled in the study and of these 46 completed the study. The reason for premature withdrawal was lost-to-follow-up. The characteristics of the patient population upon entering the study and the antidiabetic agents taken before and during the study are shown in Table 1.

Patient data at baseline prior to and after the 6 months of the study (Table 2)

**Table 2:** Patient data at baseline and after 6 months study period

	**Baseline**	**After 6 months**
N	56	46
Dropped out	N/A	10
BMI (kg/m^2^)	25.07	24.82
Blood pressure (SBP/DBP)(mmHg)	138 (9.67)/78.6 (11.5)	129.5 (10.62)/73.1 (12.0)*
Duration (y)	15.47 (10.67)	16.54 (10.88)
Laboratory data		
HbA1c (%)	6.87 (0.68)	6.86 (0.93)
FPG (mg/dl)	151.7 (47.5)	137.02 (55.22)
HWM-AND (μg/ml)	4.89 (3.16)	5.87 (3.90)*
PAI-1 (mg/dl)	27.63 (16.28)	23.17 (7.43)*
VCAM-1 (mg/dl)	715.09 (173.70)	712.06 (196.04)
U-8-OHdG (pg/ml)	11.28 (3.22)	11.65 (3.15)
Hs-CRP (mg/dl)	0.093171 (0.015391)	0.099737 (0.015174)
Blood glucose lowering treatment		
Insulin only	11	10
Insulin+OHA	7	7
OHA	32	24
Diet only	6	5

BMI, body mass index; HbA1c, glycated hemoglobin A1c; FPG, fasting plasma glucose; HMW-ADN, high molecular weight-adiponectin; PAI-1, plasminogen activator inhibitor-1; VCAM-1, vascular cell adhesion molecule-1; U-8-OHdG, urinary-8-hydroxydeoxyguanosine; Hs-CRP, high-sensitivity C-reactive protein; OHA, oral hypoglycemic agent. Data are expressed as means±s.d. or n and%. *P<0.05.

**Figure 1:**
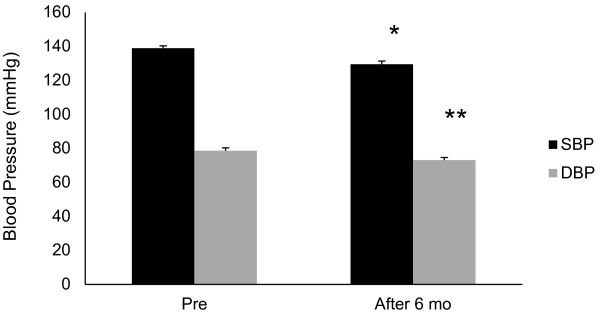
**Systolic blood pressure (SBP) and diastolic blood pressure (DPB) pre- and post-study (6 mo).** *P+0.002 for SBP and **P=0.005 for DBP.

**Figure 2:**
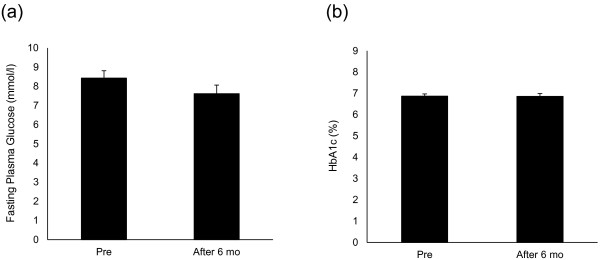
HbA1c (a) and fasting plasma glucose (b) pre- and post-study (6 mo).

**Figure 3:**
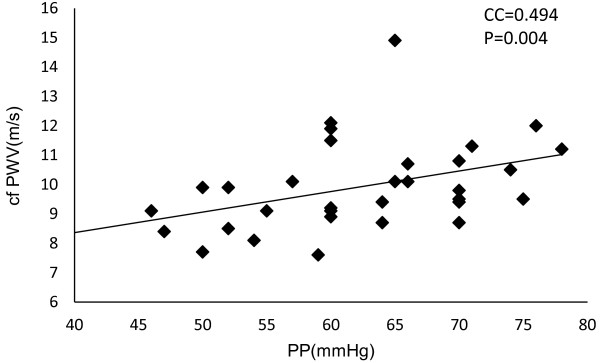
Single correlation analysis of pulse pressure (PP) and pulse wave velocity (PWV).

**Figure 4:**
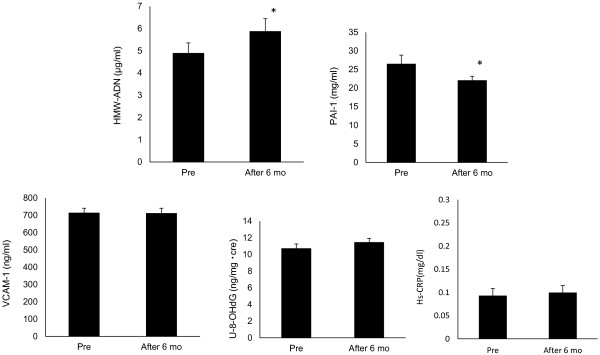
Changes from baseline of inflammatory parameters after 6 months of treatment with candesartan (*P<0.05). HMW-ADN, high molecular weight adiponectin; PAI-1, **plasminogen** activator inhibitor-1; VCAM-1, vascular cell adhesion molecule-1; U-8-OHdG, urinary 8-hydroxydeoxyguanosine.

Significant improvements from baseline values were observed in both SBP and DBP after 6 months (SBP, *P=0.002; **DBP, P=0.005, Table 2 and Figure 1). HbA1c and FPG values did not change from baseline after the 6-month treatment period (Table 2 and Figure 2). By correlation analysis we observed a significant correlation between pulse pressure and PWV (CC=0.494, P=0.004). Furthermore, multiple regression analysis showed that pulse pressure was independent of age and BMI (Table 3 and Figure 3). Significant improvements in baseline values for HMW-ADN and PAI-1 were recorded in all patients after 6 months of treatment (*P<0.05). Hs-CRP, VCAM-1 and U-8-OHdG values did not decrease from baseline after 6 months of candesartan treatment (Figure 4).

**Table 3:** Multivariate regression analysis of variables for pulse wave velocity

	**β**	**P value**
PP	0.448	0.019*
Age	−0.118	0.518(NS)
BMI	−0.028	0.875(NS)

PP, pulse pressure; BMI, body mass index.

### Relationship between HMW-ADN and PAI-1 changes and blood pressure changes

**Figure 5:**
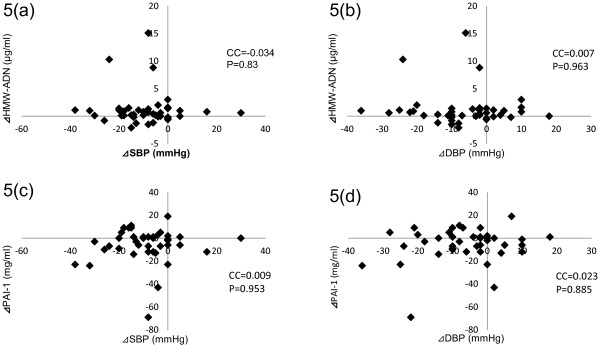
**Multilinear regression analysis of HMW-ADN and PAI-1 changes and blood pressure changes.** HMW-ADN, high molecular weight-adiponectin; PAI-1, plasminogen activator inhibitor-1; SBP, systolic blood pressure; DBP, diastolic blood pressure.

Correlation analysis was performed to establish which factors could best predict changes in patients’ HMW-ADN and PAI-1 values. Changes in blood pressure were not significant predictors of changes in the following metabolic factors in any of the patients: ⊿SBP vs. ⊿HMW-ADN CC=−0.034, P=0.83 (Figure 5a), ⊿DBP vs. ⊿HMW-ADN CC=0.007, P=0.963 (Figure 5b), ⊿SBP vs. ⊿PAI-1 CC=0.009, P=0.953 (Figure 5c), and ⊿DBP vs. ⊿PAI-1 CC=0.023, P=0.885 (Figure 5d).

### Relationship between HMW-ADN and PAI-1 changes and pulse pressure

**Figure 6:**
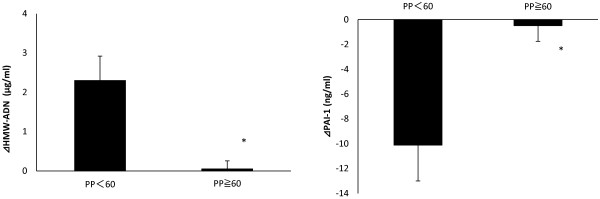
Relationship between high molecular weight-adiponectin (HMW-ADN) and plasminogen activator inhibitor-1 (PAI) changes and pulse pressure (PP).

HMW-ADN and PAI-1 values improved in patients grouped according to before-study pulse pressure < 60 mmHg compared with those grouped according to before-study pulse pressure ≥ 60 mmHg (*P<0.01,**P<0.05, Figure 6).

## Discussion

Although the antihypertension drug candesartan has been reported to control inflammation [17,18], there is little evidence about differences in effect by candesarton among each inflammatory parameter and what factors contribute to the differences. Moreover, there is the question of whether there are anti-inflammatory effects by candesartan in patients with advanced arteriosclerosis such as those with type 2 diabetes mellitus of long duration accompanied by hypertension. In this study, we analyzed the predictors showing which patients would receive the benefit of organ protection by candesartan.

### HMW-ADN and PAI-1

Although both HMW-ADN and PAI-1 values had improved significantly at the end of the study period, there was no relationship between these parameters and SBP and DBP at baseline nor did changes in SPB and DBP have any relationship between changes in these parameters. These results might indicate that candesartan improved these parameters directly and not through changes in blood pressure.

HMW-ADN is secreted from adipose tissue and has a protective effect against cardiovascular disease [19]. It was reported that HMW-ADN values change not only in the advanced stage of arteriosclerosis such with arteriosclerosis obliterans [20] but that HMW-AND is an independent prognostic factor [21].

PAI-1 is the primary physiological inhibitor of endogenous fibrinolysis that acts via inhibition of the tissue plasminogen activator (tPA) and the urokinase type activator (uPA), often leading to fibrin accumulation in basement membranes and interstitial tissues. Elevated plasma PAI-1 has been demonstrated in various subgroups as an important feature of type 2 diabetes and metabolic syndrome [22]. Plasma levels of PAI-1 were reported to predict the occurrence of a first acute myocardial infarction and reinfarction [23]. Recently, the BARI 2D Trial of subjects with a mean duration of diabetes mellitus of 10.4 years with stable coronary artery disease reported that for the older patients reducing the PAI-1 level in blood might offer an attractive strategy for decreasing cardiovascular risk [24]. The results of our study appear to support these results.

Although the mean duration of diabetes mellitus in our study was over 15 years, HMW-ADN and PAI-1 values were reduced after patients received 12 mg candesartan o.d. for 6 months. This result might partially explain the protective effect of candesartan therapy against cardiovascular disease in hypertensive patients with type 2 diabetes mellitus.

On the other hand, there is evidence that pulse pressure is an index parameter of arteriosclerosis. Pulse pressure is a marker of arteriosclerosis that can be measured simply. It is correlated with IMT and PWV, and an increase in pulse pressure has been reported to be associated with risk of onset of coronary artery disease [25,26]. It is a predictor of overall mortality in elderly persons [27]. It was also reported that pulse pressure could be a predictive factor of a cardiovascular event in persons with diabetes mellitus [28]. In healthy subjects, pulse pressure over 55 mmHg is associated with risk of a cardiovascular event [29]. Another study showed that pulse pressure over 70 mmHg presented a risk in elderly persons [30]. Among our study population, in those with type 2 diabetes mellitus of long duration, a correlation between pulse pressure and PWV was observed (CC=0.494,P=0.004). Multiple linear regression analysis suggested that pulse pressure had a relationship to PWV independent of age and BMI (Table 3).

We therefore focused attention on the relationship between changes in inflammatory parameters and changes in pulse pressure. Prior to the study, we divided patients into 2 groups, group A with high pulse pressure (66.0 mmHg ± 0.8) and group B with low pulse pressure (52.0 mmHg ± 0.8), and examined the rates of improvement in blood pressure, HMW-ADN, and PAI-1. In the total patient population, the median pulse pressure was 60.0 mmHg±1.4.

Interestingly, a significant improvement in HMW-ADN and PAI-1 was observed in the group with pulse pressure ≧60 mmHg (group A) compared with the group with pulse pressure <60 (group B). Findings were as follows: ⊿SBP: average =−9.20, P value=0.048; ⊿DBP: average =−6.61, P value=0.08; ⊿AND: average = group A 2.3 and group B 0.052 μg/ml, P value=0.005; PAI-1: average =group A −10.1 and group B −0.48 ng/ml, P value =0.012. These results suggest that diabetic patients with comparatively low pulse pressure indicating less advanced arteriosclerosis may receive benefits of improvement in inflammatory parameters such as HMW-ADN and PAI-1 by taking candesartan.

### Hs-CRP, VCAM-1 and U-8-OHdG

Hs-CRP, VCAM-1 and U-8-OHdG values did not change during this study. Also, there were no relationships between blood pressure and pulse pressure changes and Hs-CRP, VCAM-1 and U-8-OHdG.

In this study, no improvement in Hs-CRP was observed because of the following reasons. It has been reported that Hs-CRP levels are increased in patients with arteriosclerosis and other diseases that cause blood flow disturbance, and that Hs-CRP can be used as a predictor of cardiovascular events. On the other hand, it has also been reported that Hs-CRP is increased in patients with obesity and diabetes, and could be decreased by certain antidiabetic drugs. The subjects of this study had a long history of diabetes, and drugs were used in most subjects. Indeed, the Hs-CRP levels were likely below detection level in almost half of the subjects before the commencement of the study, and this might contribute to the lack of improvement of Hs-CRP. The reason might be that VACM-1 reflects early vascular endothelial dysfunction, so it increases from the onset of hypertension or diabetes mellitus [31]. The histories of diabetes and hypertension varied among the study population. Also, the study duration was too short to recognize changes in U-8-OHdG, as it could be expected to take longer for U-8-OHdG to change than the other factors evaluated. Moreover, although past clinical studies have suggested that ARB would have antioxidative and anti-inflammatory effects, most of the studies were performed in subjects that were not being treated with antihypertensive drugs. In this study, patients undergoing treatment with antihypertensive drugs, such as ARB, were included, and thus the study results might be affected by the fact that the levels of VCAM-1 and U-8-OHdG were already within a normal range prior to commencement of the study.

Alternatively, candesartan might be less effective in patients with advanced arteriosclerosis with regard to Hs-CRP, VACM-1 and U-8-OHdG. This may be why the ACCRD BP study did not find positive data for type 2 diabetic patients with hypertension from the point of view of inflammatory parameters.

It has been reported that ARB could increase adiponectin levels to a greater degree compared with calcium channel blockers and β blockers. By contrast, it has also been reported that ARB would have greater effects on VACM-1, U-8-OHdG, and PAI-1, while protecting organs, compared with calcium channel blockers in preclinical studies of diabetes. However, no difference in usefulness has been shown between the drugs in clinical studies, especially for patients with diabetes.

This study has limitations as single group pre-post study, small population, the unbalance between men and women. The results of larger clinical trials evaluating the cardiovascular protective effects including other ARBs are awaited.

## Conclusion

Candesartan improved plasma HMW-ADN and PAI-1 values but not those of plasma Hs-CRP, VACM-1 and U-8-OHdG in hypertensive patients with type 2 diabetes mellitus of long duration independently of blood pressure changes. Patients whose pulse pressure is under 60 mmHg may receive the benefits of improvement of inflammatory parameters such as HMW-ADN and PAI-1 by administration of candesartan.

## Abbreviations

HMW-ADN: High molecular weight adiponectin; PAI-1: Plasminogen activator inhibitor-1; Hs-CRP: Highly sensitive C-reactive protein; VCAM-1: Vascular cell adhesion molecule-1; U-8-OHdG: Urinary 8-hydroxydeoxyguanosine; ELISA: Enzyme-linked immunosorbent assay; PWV: Pulse pressure and pulse wave velocity; BMI: Body mass index; SBP: Systolic blood pressure; DPB: Diastolic blood pressure; FPG: Fasting plasma glucose; HbA1c: Glycated hemoglobin A1c.

## Competing interests

The authors declare that they have no competing interests.

## Authors' contributions

MS and HS conceptualized the research hypothesis and analyses, researched the data, performed all of the statistical analyses and wrote the manuscript. TH, HI, NS and YK reviewed and edited the manuscript. KT and KU assisted in conceptualizing the research question and reviewed and edited the manuscript. All authors read and approved the final manuscript.
